# VIGS as a strategy to reverse aphid wing induction by Y‐satellite RNA of cucumber mosaic virus

**DOI:** 10.1002/2211-5463.13697

**Published:** 2023-08-28

**Authors:** Hangil Kim, Chikara Masuta

**Affiliations:** ^1^ Research Faculty of Agriculture Hokkaido University Sapporo Japan; ^2^ Present address: Department of Forest Environment Protection Kangwon National University Chuncheon Korea

**Keywords:** *ABCG4*, aphid wing formation, cucumber mosaic virus, RNA silencing, virus‐induced gene silencing

## Abstract

Y‐satellite RNA (Y‐sat) of cucumber mosaic virus upregulates the expression of the aphid *ABCG4* gene, which promotes aphid wing formation. We used *ABCG4* virus‐induced gene silencing (VIGS) to prevent the wing‐induction mechanism of Y‐sat and thus inhibited aphid wing formation. Of the aphids on plants with VIGS of *ABCG4*, only about 30% had wings, and 60–70% of the winged aphids were small and likely impaired in flying ability. In addition, we showed that double‐stranded RNAs (dsRNAs) and small RNAs were transferred from the plant to the aphid to adequately silence aphid genes. Supplying *ABCG4* dsRNA by VIGS to aphids is thus a potential strategy to inhibit aphid wing formation.

## Graphical abstract

Application of virus‐induced gene silencing (VIGS) of the aphid *ABCG4* gene. We developed a strategy to inhibit aphid wing formation. *ABCG4* VIGS greatly reduced the number of winged aphids; aphids on *ABCG4* VIGS plants formed relatively shorter wings. Our results suggest that *ABCG4* silencing could be an effective strategy to reduce aphid wing formation and thus virus transmission rate.

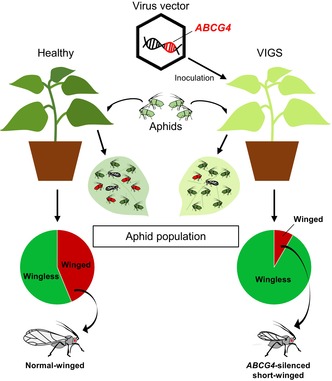

**Abbreviations**
ABCG4: ATP‐binding cassette subfamily G member 4
ChlI: magnesium protoporphyrin chelatase subunit I
CMV: cucumber mosaic virus
DCL: Dicer‐like protein
dsRNA: double‐stranded RNA
piRNA: PIWI‐interfering RNA
siRNA: small interfering RNA
sRNA: small RNA
VIGS: virus‐induced gene silencing
Y‐satellite RNA: Y‐sat

Cucumber mosaic virus (CMV) satellite RNAs (satRNAs) are 300‐ to 400‐nt, noncoding RNAs that depend on CMV for replication and encapsidation [1, 2, 3] and have little homology to CMV genomic RNAs. In general, CMV satRNAs inhibit CMV replication, thus reducing the incidence and severity of symptoms caused by lowered virus levels [4]. Among the satRNAs, Y‐satellite RNA (Y‐sat) differs from other satRNAs in causing a bright yellow leaf mosaic on tobacco [5]. The sequence in the central domain of Y‐sat (YR) has a 22‐nt continuous complementation to the mRNA of *ChlI*, a key gene for tobacco chlorophyll synthesis [6, 7]. Small interfering RNAs (siRNAs) arising from YR cause RNA silencing of *ChlI*, which impedes chlorophyll synthesis, resulting in the bright yellow mosaic. This yellow leaf color attracts aphids that transmit CMV [8]. More surprisingly, aphids infesting the yellow tobacco quickly turn red and eventually develop wings at a high frequency. We recently elucidated the mechanism of this wing induction and found that small RNAs (sRNAs) of Y‐sat directly upregulate mRNA level of the ATP‐binding cassette subfamily G member 4 gene (*ABCG4*), a key factor for wing induction in aphids [8, 9]. In other words, Y‐sat executes a surprising survival strategy in turning tobacco yellow, attracting aphids, and then inducing wing formation on the aphids, which can then fly to other plants. We then thought that if Y‐sat can induce wings on aphids by manipulating the expression of *ABCG4*, then perhaps we can prevent this induction and inhibit aphid wing formation.

We hypothesized that dsRNA or sRNA against *ABCG4* might be an effective control strategy to prevent wing formation by aphids and prevent them from flying to more plants. Various strategies to use RNA as a next‐generation alternative to chemical pesticides have been and continue to be developed. For example, double‐stranded RNA (dsRNA) or sRNA targeting specific genes of an insect pest can be synthesized and sprayed directly on the pest to eliminate it. Mitter *et al*. [10] demonstrated that simply spraying synthetic viral dsRNAs (called spray‐induced gene silencing, SIGS) on clay nanosheets is sufficient to induce antiviral resistance. Even spraying RNAs on insect vectors can reduce insect transmission of viruses. We also consider that transgenic plants that produce dsRNA or sRNA for a target gene develop resistance to the pests that have the target gene; RNA silencing for a specific target gene is induced in the insect, and the gene becomes dysfunctional. Plant viruses and pathogenic fungi can be similarly targeted [11]. For SIGS, treatment methods have been improved, and various carrier molecules for RNA delivery have been developed to extend the duration of dsRNA effectiveness in sprayed plants [12, 13, 14].

Many studies have used dsRNAs of genes important for whitefly and aphid survival to control aphids. Jain *et al*. [12] tested clay‐delivered RNA interference against genes in whitefly reducing numbers, which increased their mortality. Feng *et al*. [15] used virus‐induced gene silencing (VIGS) to silence horizontally transferred genes in aphid and whitefly, suggesting that viral dsRNA was moved from the plants to insects although they did not analyze the fate of the dsRNAs in the aphids. However, due to the nature of RNA silencing, neither of these two methods kills aphids upon exposure to the dsRNA; according to Feng *et al*. [15], silencing of aphid genes resulted in 60–70% of aphid survival rates after 1 week feeding, and thus, the aphids do not totally disappear from infested plants for a considerable time (weeks). In such a case, the control against aphid‐feeding damage is effective, but it is not expected to be very effective in controlling virus diseases because aphids can transmit viruses as soon as they start to probe a plant. Here, we considered that an effective aphid/virus control method could be developed by inhibiting aphid wing formation, thereby confining the aphids to the plants to which they are currently attached.

In this study, to inhibit wing formation, we chose to silence *ABCG4* in aphids by inserting *ABCG4* dsRNA (or sRNA) in plants using VIGS because it is a rapid, cost‐effective reverse genetics tool to silence genes in infected plants [16, 17]. We believe that SIGS and transgenic plants can also be used, but first it is important to confirm that this mechanism works as expected. However, before this kind of approach can be considered for practical application, we also need to determine whether siRNA that is synthesized in plants is transferred to aphids and actually targets *ABCG4* expression or whether the dsRNA is transferred directly to aphids and processed into sRNA in the aphid body. Here, we discuss the effectiveness of VIGS in confining aphids to the infested plant.

The inspiration for this study was to reverse the wing induction mechanism of aphids by Y‐sat. As expected, this attempt led to the rapid, frequent emergence of wingless aphids. Then, based on the results obtained in this study, we would like to propose one idea about the hidden significance of Y‐sat wing induction in aphids in the Discussion.

## Materials and methods

### Insect stocks and plant materials

A colony of peach aphid, *Myzus persicae* (Sulzer), isolated from *Brassica rapa* [8] were maintained in *N. tabacum* plants (cv. BY4) at 24 °C with 16 h light/8 h dark. The progenies from a single mother aphid were used for all the analyses. The seeds of wild‐type *Arabidopsis* Columbia (Col‐0) and the Col‐0 *dcl2dcl4* mutant (*dcl2/4*) were obtained from the Arabidopsis Biological Resource Center (ABRC, Ohio State University, Columbus, OH, USA) and grown in the growth chamber at 22 °C with 16 h light/8 h dark.

### Construction of the VIGS vectors and viral inoculation

The CMV‐A1 vector (A1) [18], which is created from RNA2 of CMV‐Y (GenBank: D12538.1) and has a truncated 2b ORF, was used as a backbone for the VIGS constructs. Two 190‐ and 239‐nt partial sequences of the *ABCG4* gene (GenBank: XM_022308174.1) were amplified using MpABCG4‐5‐190‐MluI/MpABCG4‐3‐190‐StuI and MpABCG4‐5‐239‐MluI/MpABCG4‐3‐239‐StuI primer pairs (Table S1), respectively. The amplicons were then cloned into the A1 vector in antisense orientation to construct A1‐ABCG4‐190 and A1‐ABCG4‐239 vectors. RNA 1 and 3 of CMV‐Y and the recombinant A1 vector (RNA2) were *in vitro* transcribed using T7 RNA polymerase (Takara, Shiga, Japan). The *in vitro* transcripts were mixed and used to inoculate *N. tabacum* and *Arabidopsis* plants (A1‐ABCG4). The empty A1 vector was used as a negative control.

### Quantitative real‐time RT‐PCR (qRT‐PCR)

To quantify viral RNA levels in A1‐infected and A1‐ABCG4‐infected *N. tabacum* plants, we extracted total RNA from systemically infected leaves using RNAiso Plus (Takara). After DNase A (Takara) treatment, first‐strand cDNA was synthesized using the PrimeScript RT Reagent kit (Perfect Real Time) (Takara) and oligo dT + random primers. For the qRT‐PCR, CMV‐DET‐5‐340/CMV‐DET‐3‐340 and NtEF1a‐5/NtEF1a‐3 primer pairs were used to amplify the partial sequences of CMV RNA3 and the *EF1α* gene (a reference), respectively, in the StepOnePlus Real‐Time PCR system (Applied Biosystems, Foster City, CA, USA). The values of the qRT‐PCR results were calculated using the ΔΔ*C*_T_ method. The concentrations of viral RNA were calculated based on a standard curve (Fig. S1), which was generated by qRT‐PCR using non‐inoculated tobacco total RNA mixed with *in vitro* transcribed CMV RNA3.

For analyzing the levels of aphid gene expression, total RNA was extracted from individual aphids by homogenizing the aphid in RNAiso Plus (Takara) using two beads (Ø4.5 mm) and Tomy Micro Smash MS‐100 (Tomy, Tokyo, Japan) and precipitated with 2‐propanol. After DNase A treatment, the total RNA was directly used for qRT‐PCR with One Step TB Green PrimeScript RT‐PCR Kit II (Perfect Real Time) (Takara). Q‐Ap‐ABCG4‐5‐160/Q‐Ap‐ABCG4‐3‐160 primer pair was used to quantify *ABCG4* mRNA levels. *CA‐II* mRNA levels were quantified using Mp‐CAII‐5‐150/Mp‐CAII‐3‐150 primer pair. *EF1α* was amplified using EF‐1α‐RT‐F/EF‐1α‐RT‐R primer pair as a reference. All primer sequences used for qRT‐PCRs are listed in Table S1.

### Aphid observations

For observing aphids on plants infected with A1‐ABCG4, 20 aphids [1‐day‐old apterous N(1) or N(2)] (Fig. S2) were put on non‐inoculated, A1‐infected or A1‐ABCG4‐infected plants at 10 days post inoculation (dpi). After 4 and 6 weeks, the red (alate) and the green (apterous) aphids on each plant were counted. Similarly, 12 aphids of the same age were put on non‐inoculated, A1‐infected or A1‐ABCG4‐infected Col‐0 and the Col‐0 *dcl2/4* plants, then the alate and the apterous aphids were counted after 17 days in the same aphid population. Pairwise *χ* ^2^ tests (two‐sided) were used to determine significant differences between the mean number of aphids in the alate and apterous populations for each experiment.

### Small RNA sequencing (sRNA‐seq)

In the sRNA‐seq analyses, 5′ and 3′ adaptors were ligated to sRNAs, and first‐strand cDNA was synthesized via reverse transcription. A double‐stranded cDNA library was generated by PCR enrichment, and the libraries with the 18–40‐bp insertion were sequenced with the SE50 sequencing system (MGI). The raw reads of sRNAs were trimmed and filtered (> Q20) with Trim Galore v.0.6.7 (https://www.bioinformatics.babraham.ac.uk/projects/trim_galore/) to obtain only high‐quality reads, then mapped to the genome sequences of *N. tabacum*, *M. persicae* and the recombinant CMV using Bowtie2 v2.2.5 (https://bowtie‐bio.sourceforge.net/bowtie2/index.shtml). The mapped sRNA reads were extracted using SAMtools v1.14 (http://www.htslib.org/). Size distribution of the aligned sRNA reads was analyzed using language C in Microsoft Visual Studio 2019 v16.11.21 (Microsoft) as described by Jayasinghe *et al*. [8].

### Northern blot analysis of sRNAs

High‐molecular weight RNAs in the total RNA were first precipitated using 20% polyethylene glycol #6000, then low‐molecular weight (LMW) RNAs were precipitated from the supernatant using ethanol. The LMW RNAs were separated by PAGE in a 15% polyacrylamide gel and blotted onto an Amersham Hybond‐N^+^ membrane (GE Healthcare, NJ, USA). Northern blot hybridization was then done as previously described [6]. The 3′ end region of CMV RNA3 was PCR‐amplified using primer pairs of CMV‐DET‐5‐340 and T7‐Y3‐3 (Table S1) containing the T7 promoter sequence and used for *in vitro* transcription. An antisense *in vitro* transcript labeled with digoxigenin (DIG) was used as a probe to detect CMV sRNAs. The bands were detected using anti‐digoxigenin‐AP Fab fragments and CDP‐Star (Roche, Mannhelm, Germany).

## Results and Discussion

### Construction of the CMV vector to silence *ABCG4*

**Fig. 1:**
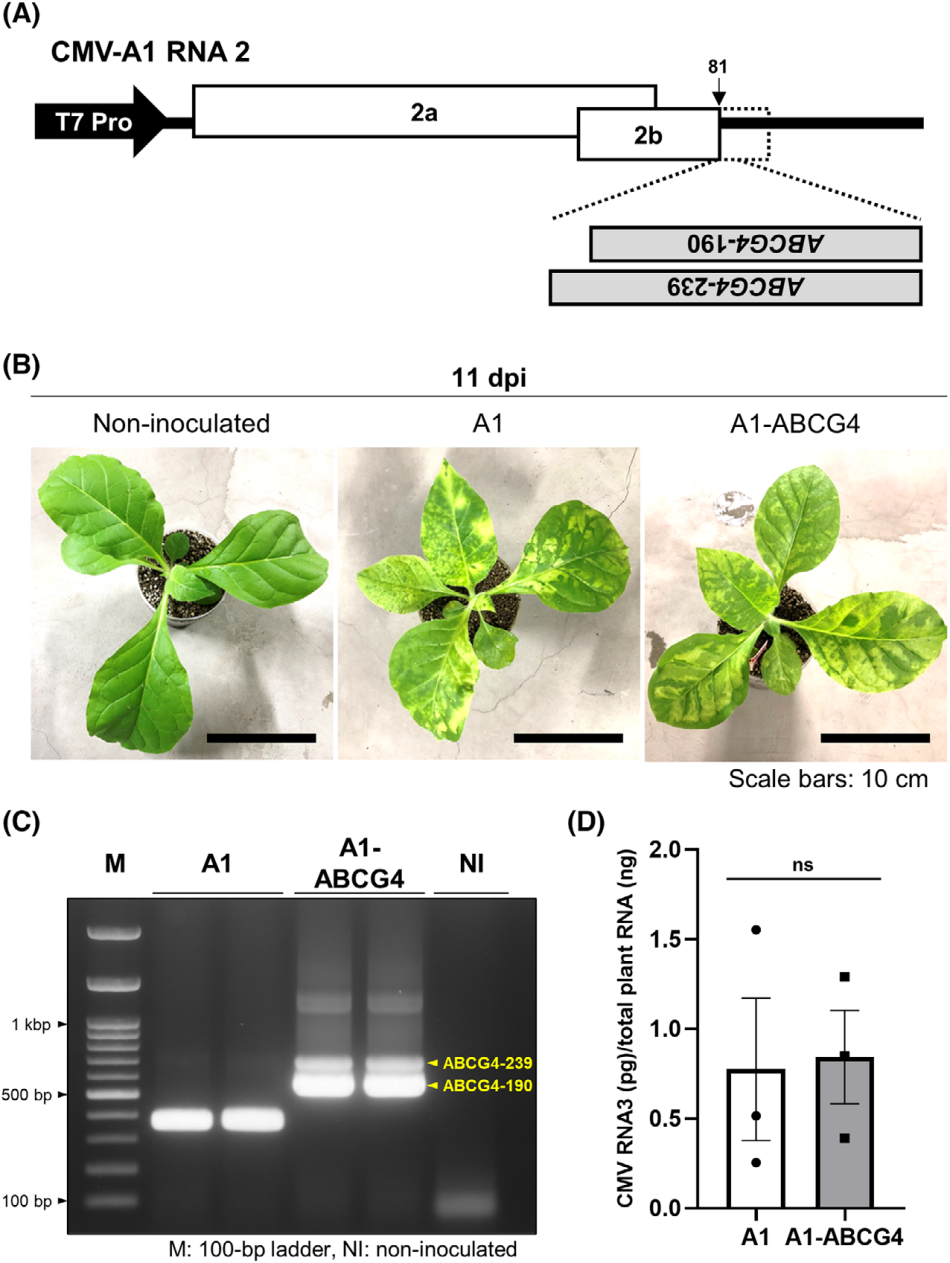
Construction of the cucumber mosaic virus (CMV) vector for *ABCG4* silencing. (A) Schematic structure of the recombinant CMV‐A1 vector. Two partial sequences (190‐ and 239‐nt fragments) of the *ABCG4* gene were cloned into the A1 vector in the multiple cloning site [18] in reverse‐complementary orientation to generate A1‐ABCG4‐190 and A1‐ABCG4‐239. (B) Mild mosaic on tobacco plants (*N. tabacum* L. cv. BY4) 11 days post inoculation (dpi) with A1 or A1‐ABCG4. Scale bars: 10 cm. (C) RT‐PCR detection of the 190‐ and 239‐nt vector‐inserted *ABCG4* sequences in tobacco plants infected with A1‐ABCG4. Primer pair 2b‐5‐up/R2‐2814R2 (Table S1) were used. (D) Mean (± SEM) CMV RNA levels in A1‐ and A1‐ABCG4‐inoculated tobacco plants at 11 dpi quantified by qRT‐PCR using primer pair CMV‐DET‐5‐340/CMV‐DET‐3‐340 (Table S1). As a reference, *EF1α* was amplified using primer pair EF1a‐5/EF1a‐3. The concentration of viral RNA was calculated based on a standard curve in Fig. S1. Mean values of CMV RNA concentration (± SEM) for the two treatments were analyzed for significant differences using a two‐sided Student's *t*‐test (*n* = 3) (*P* > 0.05). ns, not significant.

To test whether *ABCG4* silencing indeed inhibits aphid wing formation, we used VIGS with the CMV vector. The 190‐ and 239‐nt fragments of the *ABCG4* gene were inserted into the CMV‐A1 vector [18] in antisense orientation to create A1‐ABCG4‐190 and A1‐ABCG4‐239, respectively (Fig. 1A). By 11 days post inoculation (dpi) of tobacco plants with *in vitro* transcripts of the recombinant clones and the clones of CMV RNAs 1 and 3, mild yellowing was apparent on systemically infected leaves of the A1‐ and the A1‐ABCG4‐inoculated plants (Fig. 1B). As shown in Fig. 1C, the 190‐ and 239‐nt inserts were still maintained in the viral genome. The level of the recombinant virus in the upper tissues was almost similar to that of the control A1 vector (~ 0.8 pg of CMV RNA3 per 1 ng total plant RNA) (Fig. 1D). This good propagation of the recombinant virus guarantees that abundant viral dsRNAs and sRNAs were generated by VIGS in the infected plants.

### VIGS ABCG4 reduced the winged aphid population

**Fig. 2:**
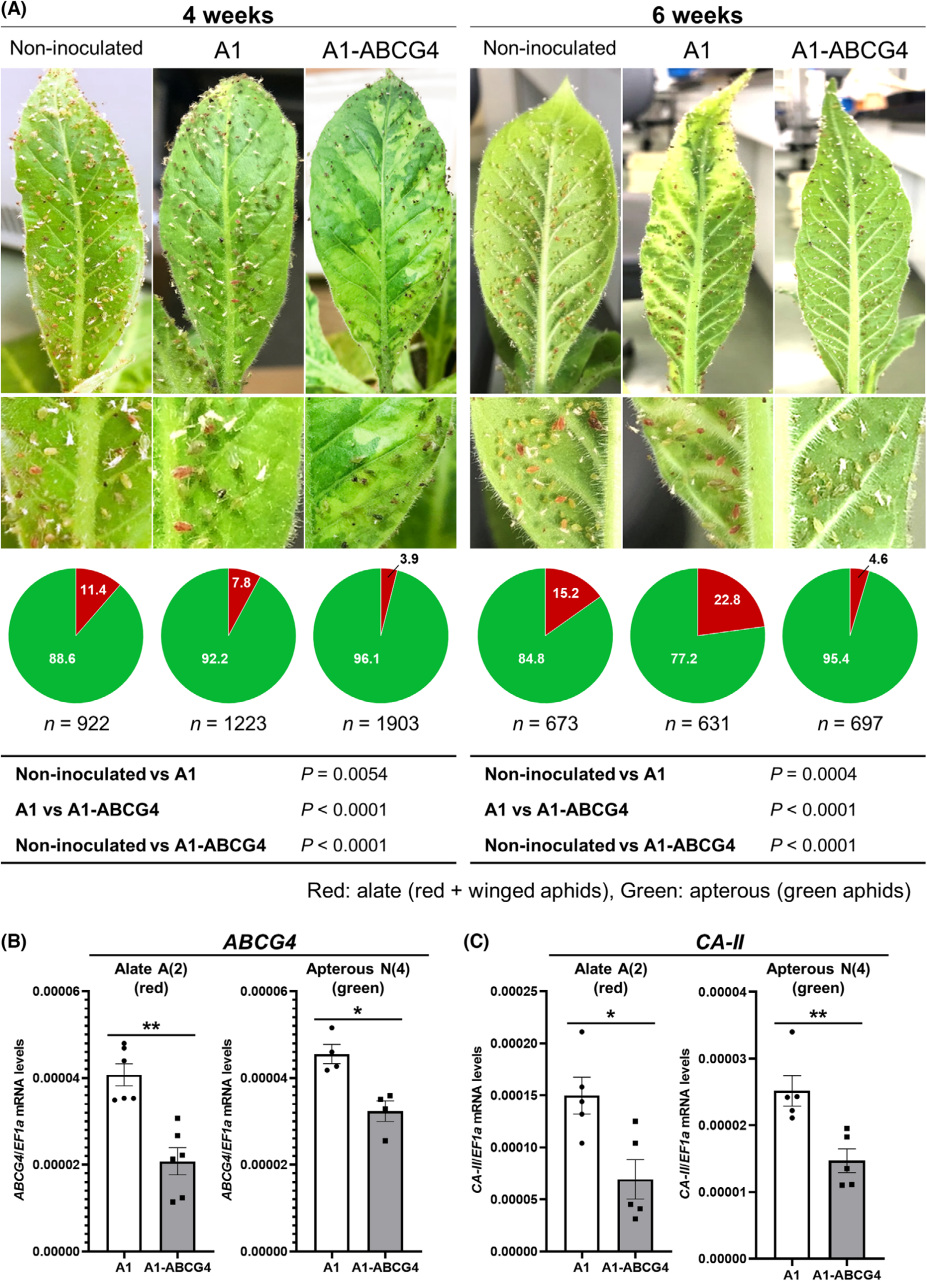
Effect of *ABCG4* VIGS in tobacco plants on aphid wing formation. (A) Mean percentages of alate (red and winged aphids) and apterous (green aphids) morphs on non‐inoculated, A1‐infected and A1‐ABCG4‐infected tobacco plants at 4 and 6 weeks after placing aphids on each plant. Pairwise *χ* ^2^ tests (two‐sided) were performed for the statistical analyses. Sample size for counted aphids and *P* values are shown below the circle graphs. (B) *ABCG4* expression levels in alate A(2) (*n* = 6) and apterous N(4) (*n* = 4) aphids that fed on A1‐ and A1‐ABCG4‐infected tobacco plants. (C) *CA‐II* expression levels in alate A(2) (*n* = 5) and apterous N(4) (*n* = 5) aphids fed on A1‐ and A1‐ABCG4‐infected tobacco plants. (B, C) The *EF1α* gene was amplified as a reference. Means (± SEM) for *ABCG4*/*EF1α* (B) and *CA‐II*/*EF1α* (C) mRNA levels were analyzed using a two‐sided Student's *t*‐test (*P < 0.05, **P < 0.01).

We previously found that when the green aphid body changes to red (red nymph), the red aphid soon develops wings (alate adult), while a green aphid (green nymph) develops into a wingless adult (apterous adult) [8]. We also found that ABCG4 plays an important role in the mechanism by which CMV Y‐sat promotes aphid wing formation [8]. Here, in our test of VIGS in tobacco plants for suppressing the expression of *ABCG4* in aphids and inhibiting aphid wing formation, our primary interest was whether the viral dsRNA or siRNA produced in tobacco is translocated into the aphids and induces silencing of the target gene in the aphids. After the apterous aphids were placed on non‐inoculated, A1‐infected, or A1‐ABCG4‐infected plants, the percentage of red, winged aphids in the total aphid population was significantly lower on A1‐ABCG4‐infected tobacco than on non‐inoculated or A1‐infected tobacco (Fig. 2A). To evaluate the possible effect of wounding by mechanical inoculation on aphid wing formation, the red/green aphid ratios were compared between untouched non‐inoculated and mock‐inoculated plants at 2 and 3 weeks after infestation, and we found that there was no significant difference (Fig. S3). These results suggest that wing formation on aphids was inhibited by VIGS against *ABCG4* and that the VIGS in the plants actually functions in the aphids; thus, *ABCG4* dsRNA, sRNA or both must have been transferred to aphids from tobacco. Confirming that *ABCG4* was indeed silenced by VIGS in the aphids, the level of *ABCG4* mRNA dropped by about half in the aphids on A1‐ABCG4‐infected tobacco compared to those on the control A1‐infected tobacco (Fig. 2B). *ABCG4* expression was also reduced in aphids regardless of growth stage [e.g., alate A(2) (red) or apterous N(4) (green)] (Fig. S2). Considering that Shang *et al*. [9] previously demonstrated that *ABCG4* in another aphid species (*Acyrhosiphon pisum*) was essential for various wing‐related network genes, *ABCG4* silencing may be a very effective way to inhibit aphid wing formation.

**Fig. 3:**
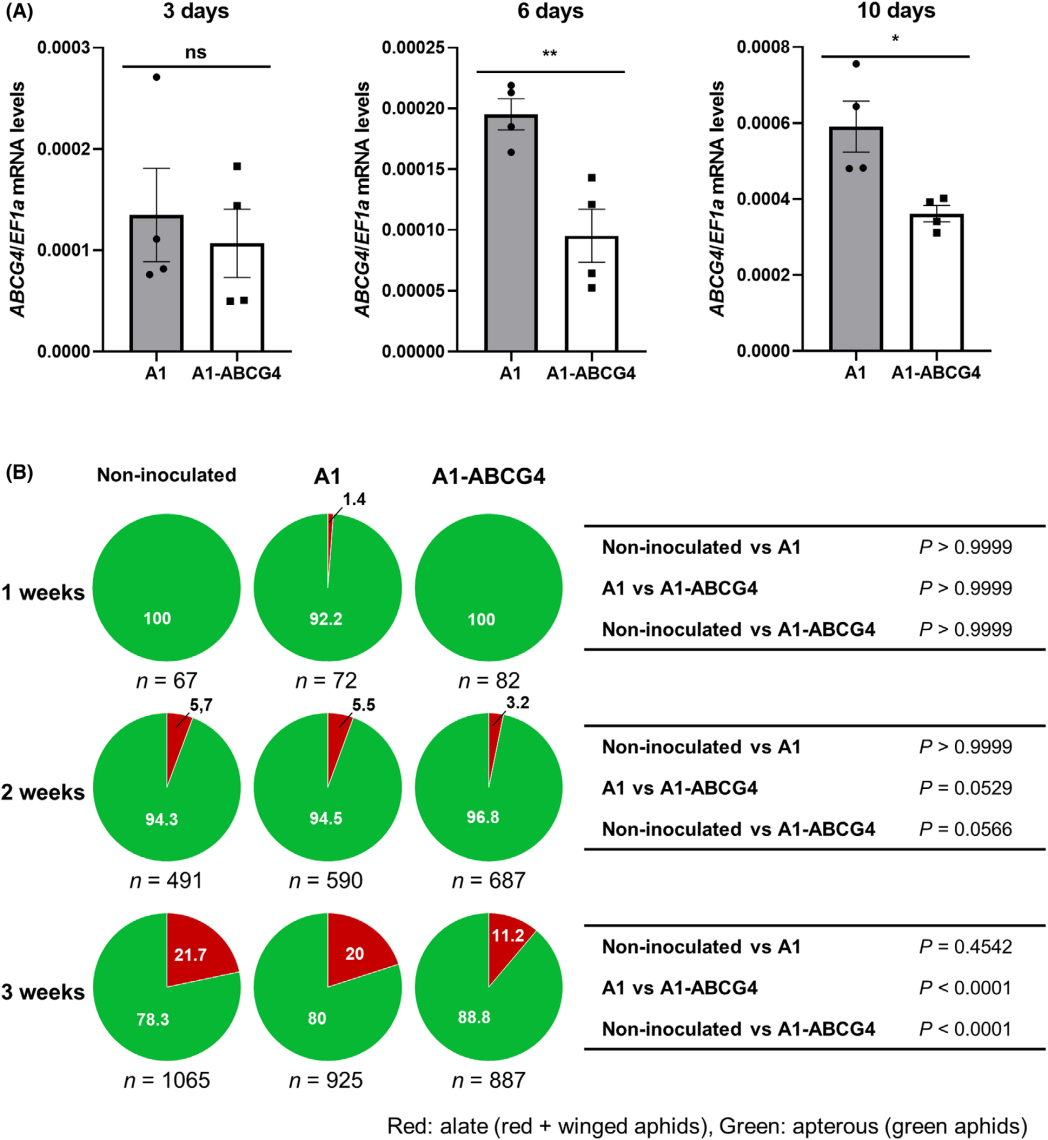
Estimation of aphid minimum feeding time required for *ABCG4* silencing and suppression of wing formation. (A) Time‐course qRT‐PCR to evaluate *ABCG4* silencing in aphids feeding on A1‐ABCG4‐infected tobacco. The aphid individuals that had been fed on A1‐ and A1‐ABCG4‐infected tobacco plants for 3, 6, and 10 days were analyzed. The qRT‐PCRs were conducted as described in Fig. 2 legend. Mean (± SEM) values were analyzed for significant difference using Student's *t*‐test (*n* = 4) (*P < 0.05, **P < 0.01). (B) Mean percentages of alate and apterous aphids on non‐inoculated, A1‐infected and A1‐ABCG4‐infected tobacco plants at 1–3 weeks after placing aphids on each plant are shown in pie charts. Pairwise *χ* ^2^ tests (two‐sided) were performed for the statistical analyses (right panel).

To estimate the minimum feeding time for *ABCG4* silencing, we performed time‐course qRT‐PCR of *ABCG4* in the aphids fed on A1‐ABCG4‐infected tobacco plants. As a result, at day 3, there appeared to be a mix of individuals with and without reduced *ABCG4* expression, but by day 6, all individuals reduced *ABCG4* expression by about half compared to the aphid individuals placed on the control, A1‐infected tobacco (Fig. 3A). Therefore, between days 3 and 6, *ABCG4* silencing seemed to have occurred in all individuals placed on the A1‐ABCG4‐infected tobacco. To evaluate how long feeding time is required until we can actually see reduced winged aphid population, we observed the red/green aphid ratio in a time‐course manner. At 1 week after aphid placement, little red aphid appeared (Fig. 3B), However after 2 weeks, we observed a reduced number of red nymphs in the A1‐ABCG4‐infected plants (Fig. 3B). In this observation, it must be taken into account that nymphs are newly added one after another by monogenesis every 5–7 days; the feeding time of these new nymphs is thus different from that of the parent insects initially placed on the tobacco. Given that red nymphs start to appear around 2 weeks after original aphid placement when the population size becomes large enough to induce winged aphids, we consider that the *ABCG4* VIGS can effectively reduce winged aphid population.

### Does the Y‐sat‐induced upregulation of *ABCG4* affect the expression of *CA‐II* ?

Recently, winged aphids were shown to have a higher virus transmission efficiency than wingless aphids, which was attributed to higher expression of the carbonic anhydrase II (*CA‐II*) gene in winged aphids than in the wingless [19]. As noted earlier, winged aphids are also more efficient than the wingless at virus transmission, which is associated with greater expression of *CA‐II* [19]. Here, in aphids that had fed on A1‐ABCG4‐infected tobacco plants to induce *ABCG4* VIGS, the expression of *CA‐II* was reduced to about half that of the control aphids that had fed on A1‐infected tobacco (Fig. 2C). Thus, VIGS of *ABCG4* alone can also effectively suppress virus transmission by aphids to other plants.

This result provides new insights into the survival strategy of Y‐sat [8]. We presume that the Y‐sat‐induced upregulation of *ABCG4* expression, which induces wing formation, results incidentally in an increase in *CA‐II* expression. To verify this hypothesis, we compared *CA‐II* expression between aphids that fed on CMV‐ and [CMV + Y‐sat]‐infected tobacco plants and found that *CA‐II* expression was enhanced by Y‐sat infection (Fig. S4). Because Y‐sat multiplication greatly lowers CMV levels, virus transmission rate by aphids is generally reduced. However, Y‐sat seems to benefit from the phenomenon of higher virus transmission efficiency in winged aphids.

### *ABCG4* silencing reduced the size of alate aphids

**Fig. 4:**
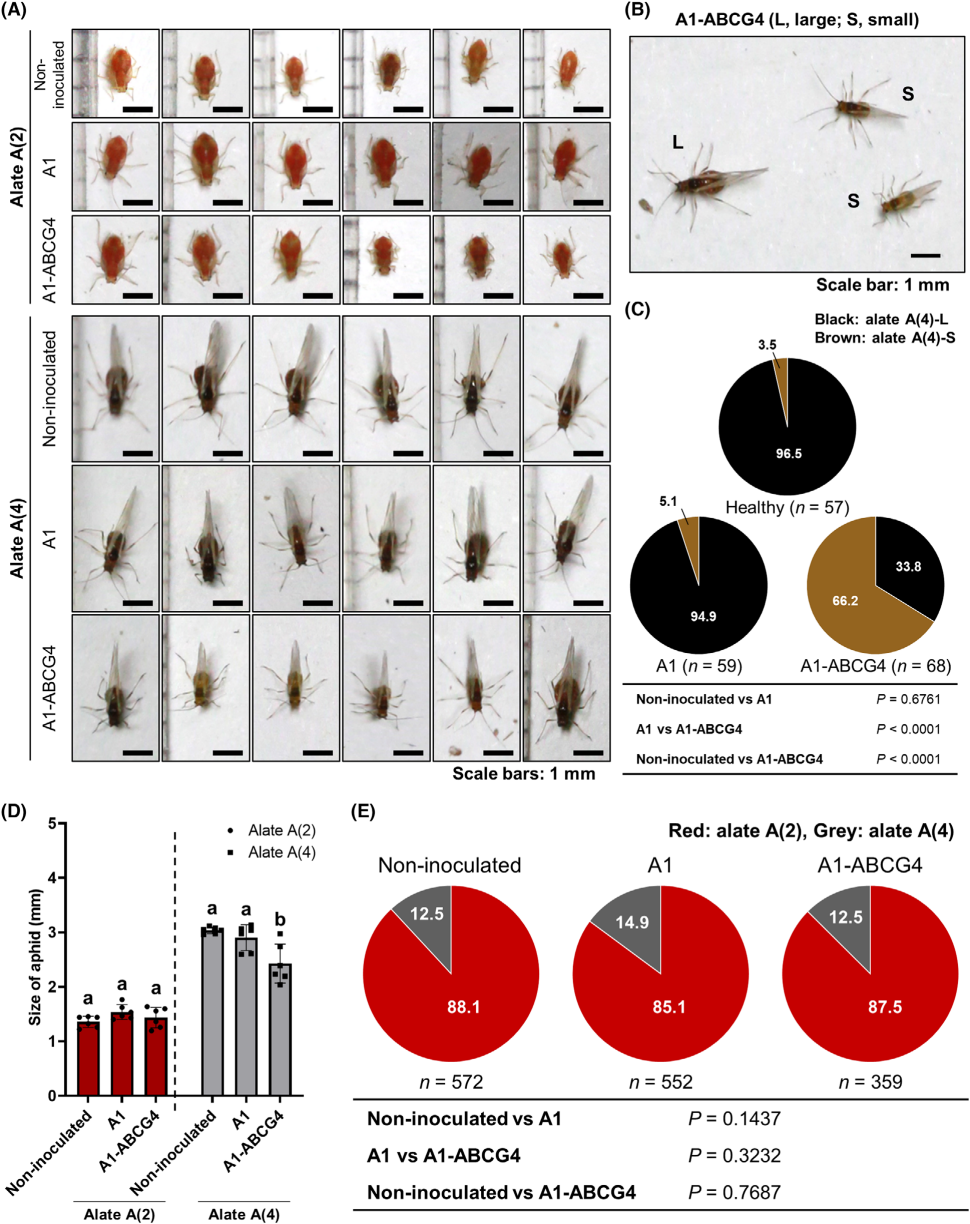
Effect of *ABCG4* VIGS on aphid development. (A) Morphs of early adult [alate A(2)] and late adult [alate A(4)] aphids that fed on non‐inoculated, A1‐infected and A1‐ABCG4‐infected tobacco plants. Scale bars: 1 mm. (B) Different sizes of alate A(4) aphids on A1‐ABCG4‐infected tobacco. (C) Percentages of large alate [Alate A(4)‐L] and small alate [Alate A(4)‐S] individuals on non‐inoculated, A1‐infected and A1‐ABCG4‐infected plants. Pairwise *χ* ^2^ tests (two‐sided) were done as described in Fig. 2A legend. (D) Sizes of individual alate A(2) and alate A(4) aphids fed on non‐inoculated, A1‐infected and A1‐ABCG4‐infected plants. Mean aphid sizes (mm) (± SEM) were analyzed for differences among treatments using Tukey's multiple comparison test (*n* = 6); different letters above the bars indicate a significant difference between means (*P* < 0.05). (E) Proportion of early adult [alate A(2)] and late adult [alate A(4)] aphids that fed on non‐inoculated, A1‐infected and A1‐ABCG4‐infected tobacco plants. Pairwise *χ* ^2^ tests (two‐sided) were done as described in Fig. 2A legend.

Shang *et al*. [9] previously reported that malformed wings developed on aphids that had fed on plants in which artificially synthesized dsRNA of *ABCG4* had been introduced into the petiole of a detached leaf. When we tested whether dsRNA of *ABCG4* in our VIGS system altered the wing or body morphology of the aphids grown on A1‐ABCG4‐infected plants, all aphids had apparently normal wings. However, when we compared the alate A(4) stage aphids grown on A1‐ABCG4‐infected plants with average‐sized aphids (L‐type) grown on the control A1‐infected plants, a high frequency of the alate‐stage aphids were short‐winged aphids (S‐type); this difference was not observed for the alate A(2) stage aphids (Fig. 4A,B). Measurements of winged aphid bodies at the alate A(4) stage showed that the frequency of S‐type aphids that fed on non‐inoculated plants was 3.5% vs 5.1% on A1‐infected plants, but 66.2% for those on A1‐ABCG4‐infected tobacco (Fig. 4C). As shown in Fig. 4D, the body size was 15–20% shorter at the alate A(4) stage. Once aphids turn red, all of them seemed to develop wings, suggesting that ABCG4 functions in an early stage of wing development (Fig. 4E). These results suggest that the reduced expression of *ABCG4* in the aphids also inhibits aphid development during wing formation.

Thus, although not obviously deformed, the shorter wings may affect flight distance because aphids fly mainly by gliding rather than moving their wings [20]; the shortened wings may also negatively affect their feats of dispersal through wind currents [21]. As to why the reduced expression of *ABCG4* leads to dwarfing of aphid body size, nutrient intake from the plant could be affected because ABCG4 has been implicated in promoting the secretion of several insulin‐like peptides (ILPs) to regulate the insulin/insulin‐like growth factor signaling (IIS) pathway (a conserved nutrient sensor mechanism) [9, 22]. We do not know why our system did not generate any wing deformities as seen by Shang *et al*. [9], but differences such as the method of dsRNA supply or aphid species could lead to differences such as the amount of dsRNA ingested.

### How do siRNAs target *ABCG4* in aphids on the VIGS plants?

How does *ABCG4* dsRNA or siRNA generated in the plant induce RNA silencing against *ABCG4* in the aphids? Because CMV does not multiply in aphids, three mechanisms seem possible: (1) Plant‐generated *ABCG4* dsRNAs or siRNAs are absorbed by and function in that form in the aphid. (2) Plant‐generated dsRNA is absorbed by the aphid, cleaved into siRNAs by aphid Dicers, and function in that form. (3) Both (1) and (2) occur. To verify which case is correct, we decided to use *Arabidopsis* because the *Arabidopsis* DCL2/DCL4 double‐knockout mutant (*dcl2/4*) was available. In *dcl2/4 Arabidopsis*, 21‐ and 22‐nt siRNAs are not synthesized [23]. Therefore, by analyzing whether the extracted aphid RNA contains 21−/22‐nt siRNA, we can determine whether *ABCG4* dsRNA is transferred from the plant to the aphid.

**Fig. 5:**
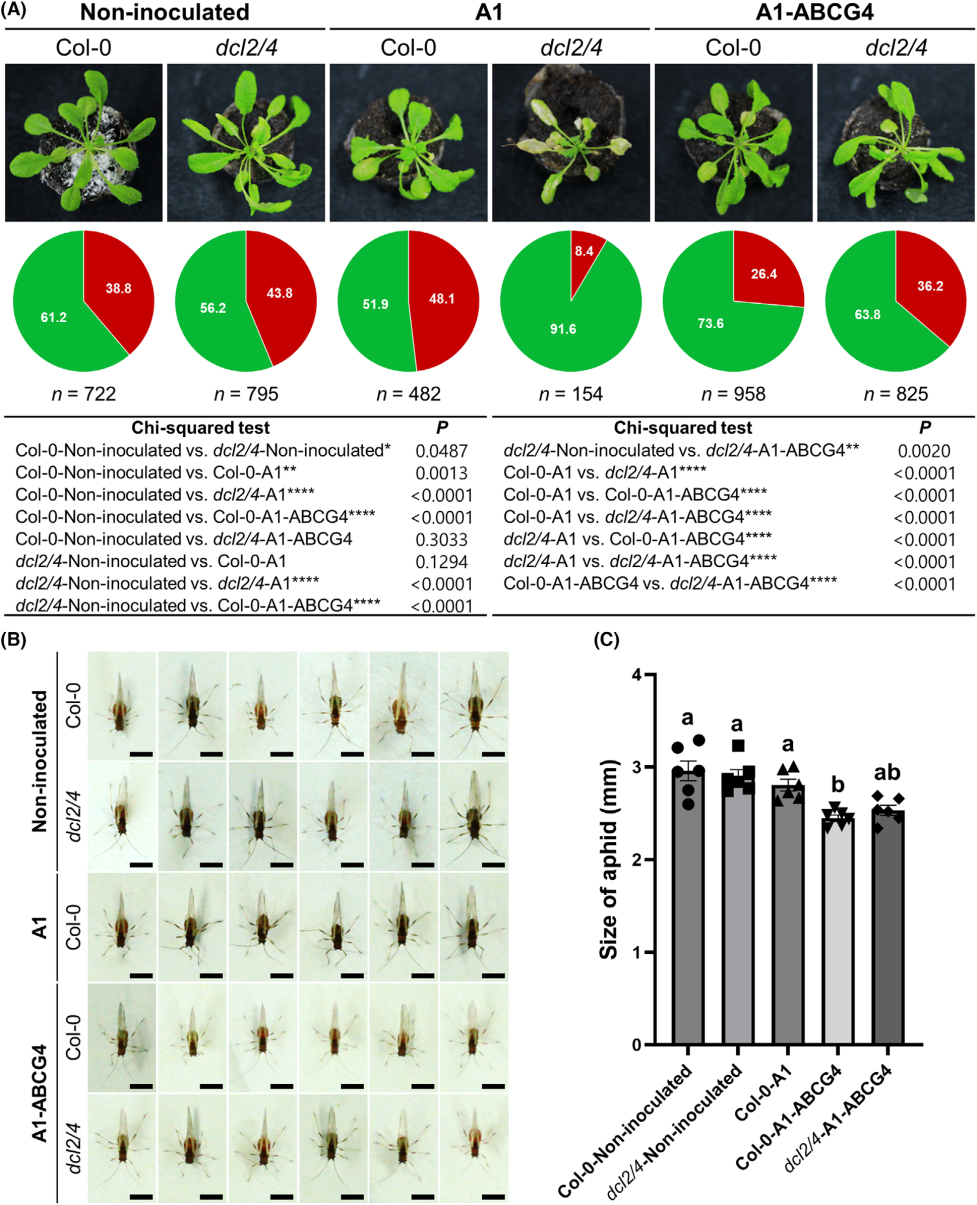
Effect of *ABCG4* VIGS in *Arabidopsis* plants on aphid wing formation. (A) Mean percentages of apterous and alate aphids on non‐inoculated, A1‐infected and A1‐ABCG4‐infected wild‐type *Arabidopsis* (Col‐0) and the Col‐0 *dcl2/4* mutant plants. Twelve aphids (1‐day‐old) were put on each plant, and the alate and apterous aphids were counted after 17 days. Images of non‐inoculated and infected plants were taken at 24 dpi. Sample sizes for counted aphids are shown below each circle graph. Pairwise *χ* ^2^ tests (two‐sided) were done as described in Fig. 2A legend. (B) Morphs of alate A(4) aphids that fed on non‐inoculated, A1‐infected and A1‐ABCG4‐infected Col‐0 and the Col‐0 *dcl2/4* mutant 17 days after aphids were placed on plants. Scale bars: 1 mm. (C) Sizes of individual alate A(4) aphids on non‐inoculated, A1‐infected and A1‐ABCG4‐infected Col‐0 and the Col‐0 *dcl2/4* plants. Mean aphid sizes (mm) (± SEM) were analyzed by Tukey's multiple comparison test (*n* = 6). Different letters above the bars indicate significant difference (*P* < 0.05).

For this purpose, *Arabidopsis* plants (Col‐0 and *dcl2/4*) were inoculated with A1 or A1‐ABCG4, then 10 days later, 12 aphids (1‐day‐old) were placed on the plants, and 17 days later, the frequency and phenotype of green and red aphid alates were recorded. Disease symptoms on A1‐infected *dcl2/4* plants were much more severe than on the non‐inoculated plants, and many individuals had lethal necrosis (Fig. 5A). The red/green ratio was clearly lower for aphids on A1‐ABCG4‐infected Col‐0 plants than on non‐inoculated and A1‐infected Col‐0 plants (Fig. 5A). These results are consistent with the results on tobacco in Fig. 2, but the percentages of red aphid were relatively higher in *Arabidopsis* than those in tobacco plants (Figs 2A and 5A); it may be due to the aphid propagation in a smaller space, and thus the aphids were under higher population stress, which would affect aphid wing formation. The red/green ratio for aphids on A1‐ABCG4‐infected *dcl2/4* plants was also lower than on non‐inoculated *dcl2/4* plants and on A1‐infected Col‐0 plants, indicating that *ABCG4* VIGS actually occurred in the aphids on A1‐ABCG4‐infected *dcl2/4* plants although the *dcl2/4* plants had impaired RNA silencing. However, the A1‐infected *dcl2/4* plants were not comparable to the other plants because aphid growth on A1‐infected *dcl2/4* plants was greatly inhibited by the lethal necrosis of the plants (Fig. 5A and Fig. S5), perhaps because RNA silencing does not work against the virus in *dcl2/4* plants, resulting in much higher viral replication and thus more severe symptoms. On the other hand, in A1‐ABCG4 plants, the presence of the insert sequence may have suppressed virus multiplication to some extent. When we measured body sizes of aphids grown on *Arabidopsis*, winged aphids on A1‐ABCG4‐infected plants were significantly smaller than those on non‐inoculated or A1‐infected plants (Fig. 5B,C). Again, these results agree well with those on tobacco in Fig. 2. Based on these results, there seem to be few differences in the VIGS against *ABCG4* between the two plant species.

We next extracted RNA from A1‐ABCG4‐infected plants and aphids attached to those plants for sRNA‐seq analysis. When we examined the origin of sRNA in tobacco and aphid RNA, the majority (56.14%) of CMV vsiRNAs that resulted from VIGS were detected in tobacco; < 0.02% were detected in the aphids on tobacco (Fig. S6). On the other hand, a significant amount (10.95% of the total) of sRNA from the tobacco genome appears to be transferred to the aphid (Fig. S6). The reason is not clear, but perhaps the aphids can discriminate the sequences of the sRNAs taken into their bodies. The percentage (74.75% of total sRNA) of CMV vsiRNAs in the A1‐ABCG4‐infected *Arabidopsis* was significantly higher than that of tobacco (43.86%; Fig. S6). Because vsiRNA levels are considered to be important to determine VIGS efficiency in aphids, we presume that the effect of *ABCG4* VIGS on aphid wing formation may be different depending on the plant used. To clearly demonstrate the factors to affect such VIGS efficiency in aphids, we would need further experiments [e.g., the analyses of viral RNA (vsiRNA) levels in plants and aphids, and the RNA absorption mechanism by aphid cells].

**Fig. 6:**
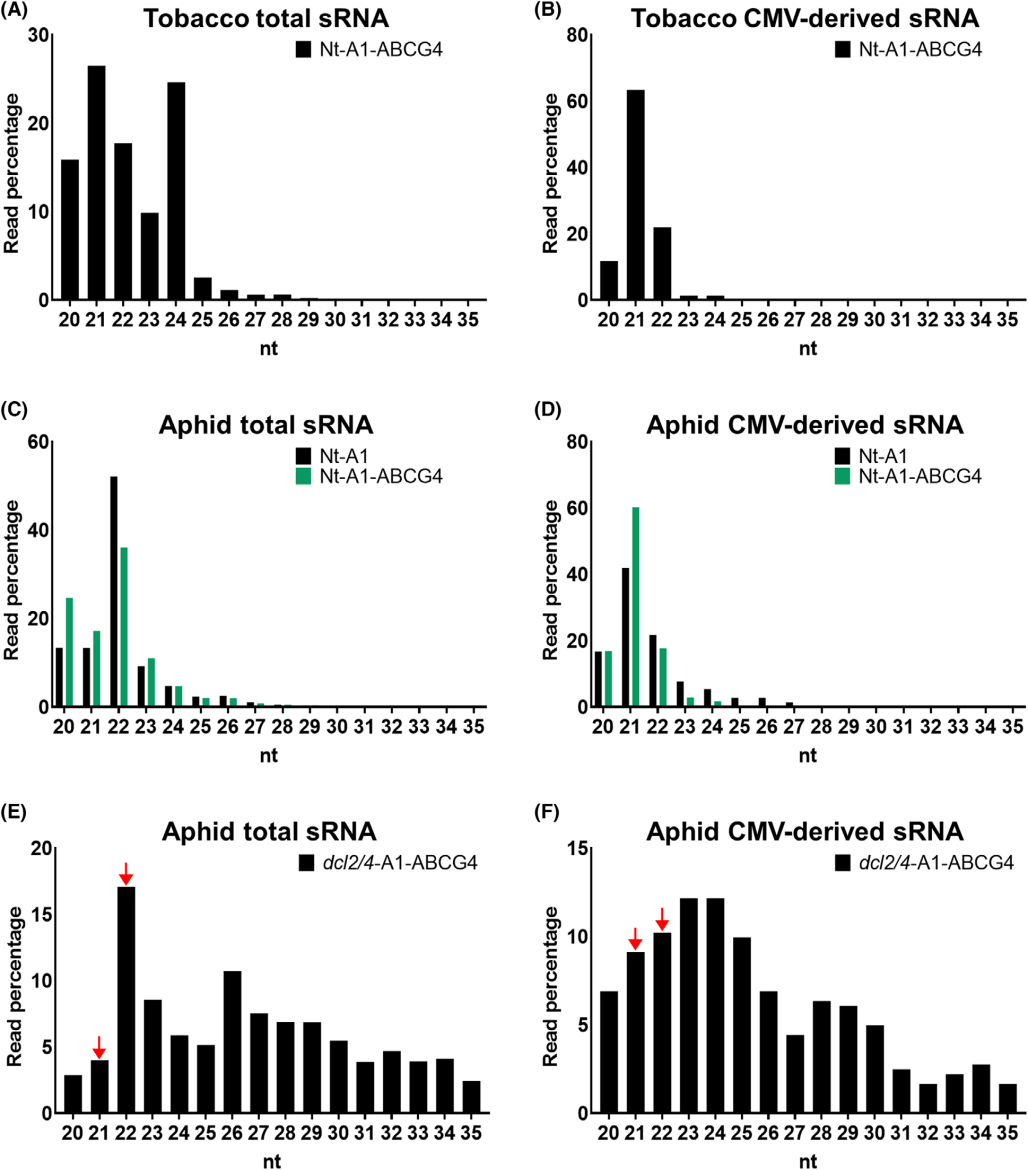
Size distribution of sRNAs isolated from aphids and aphid‐infested, A1‐infected and A1‐ABCG4‐infected tobacco and *Arabidopsis* plants. The 20–35‐nt sRNA reads were extracted from the sRNA‐seq data and used for the analyses. (A, B) Size distribution of tobacco genome‐derived (A) and CMV‐derived (B) sRNAs from A1‐ABCG4‐infected tobacco plants. (C, D) Size distribution of aphid genome‐derived (C) and CMV‐derived (D) sRNAs from the aphids on A1‐infected and A1‐ABCG4‐infected tobacco. (E, F) Size distribution of aphid genome‐derived (E) and CMV‐derived (D) sRNAs from aphids on A1‐ABCG4‐infected Col‐0 *dcl2/4* mutant. Red arrows indicate the 21‐ and 22‐nt sRNAs possibly transported from the aphid‐infested *dcl2/4* plants to the aphids that fed on them.

A subsequent analysis showed that the size distribution of the sRNAs from the tobacco genome ranged from 20‐ to 24‐nt, which appear to be the typical sRNAs generated by DCL1‐4 in tobacco (Fig. 6A), and the CMV vsiRNAs were distributed all over the CMV genome. On the other hand, CMV vsiRNAs peaked mainly at 21‐ and 22‐nt, suggesting that they were generated by DCL2 and DCL4, targeting viral RNA (Fig. 6B). In aphids, sRNAs derived from the aphid genome formed a sharp peak at 22‐nt, while CMV vsiRNA had a peak at 21‐nt (Fig. 6C,D). Therefore, to confirm whether the 21‐nt vsiRNAs of CMV were plant‐derived or generated in the aphid, we did a similar experiment using *Arabidopsis dcl2/4* mutant instead of tobacco. We first confirmed by northern blot analysis that the *dcl2/4* plant produced mostly 24‐nt siRNA (Fig. S7). We clearly observed a peak at 22‐nt for sRNAs derived from the aphid genome (Fig. 6E), similar to the result in tobacco, but detected a broad peak at 21–25‐nt for CMV vsiRNAs (Fig. 6F). Figure 6E,F show relatively longer sRNAs, between 25‐ and 35‐nt, albeit at much lower amounts; these sRNAs with higher molecular weights may be PIWI‐interacting RNAs (piRNAs). In insect RNA silencing, the dicer enzymes, DCR1 and DCR2 generates 21–23‐nt sRNAs (mainly miRNA), and 21–22‐nt sRNAs, respectively [24]. In addition, 23–31‐nt piRNAs are frequently observed; unlike other classes of sRNAs, they are processed from ssRNA precursors and thought to be produced primarily as a viral resistance response [25, 26]. When aphids were grown on tobacco (Fig. 6C,D), 21–22‐nt sRNAs were actively produced by DCR1/DCR2, although piRNAs were also synthesized to some extent. On the other hand, when aphids were grown in *Arabidopsis* (Fig. 6E,F), DCR1/DCR2 activity was present but somewhat lower compared to the levels in tobacco. The reason is not clear, but differences between the tobacco and *Arabidopsis* plants might affect RNA silencing in aphids. As reported by Sattar *et al*. [27], just one gene difference in melon plants can affect the levels of siRNAs and piRNAs; in aphids on melons with and without the *Vat* gene for resistance to virus transmission, aphids that fed on melons with *Vat* had lower levels of 21–22‐nt siRNAs and much higher levels of piRNAs.

Importantly, given the inability of *Arabidopsis dcl2/4* plants to synthesize 21‐ and 22‐nt sRNAs [28], the 21‐ and 22‐nt sRNAs detected in aphids were likely generated by the aphid Dicer(s) (possibly DCR1/DCR2) from dsRNA transferred from the plant to the aphid. The sharp peak at 22‐nt detected in sRNAs derived from the aphid genome (Fig. 6E), as found for several other insects [28, 29, 30], suggests that like the other insects, the aphids have a Dicer species that specifically generates 22‐nt sRNA. Furthermore, as shown in Fig. 6F, the size distribution of the CMV vsiRNAs is completely different from the distribution of those derived from the aphid genome. Thus, in the aphids, viral dsRNA (even if it is a plant virus) may be processed by a different dicer from the one that produces the aphid genome‐derived sRNA.

## Conclusion

We demonstrated that aphid wing formation can be inhibited by VIGS of the aphid gene *ABCG4*. The implication is that wingless individuals can be restricted to their present plant. Even if any genes essential for aphid survival are targeted by RNA silencing via VIGS, it cannot kill the individual as quickly as a chemical pesticide; the aphid can still transmit viruses to other plants until they die. Containing wingless aphids to one plant would be the best way to control the virus diseases. We here demonstrated that *ABCG4* silencing could efficiently reduce winged aphid population and produce the alate adults with short wings, which would reduce aphid dispersal rate. Furthermore, if *ABCG4* VIGS can be implemented, long‐term aphid control would be possible simply by pre‐infecting plants with attenuated viruses. However, because of the reluctance to use recombinant viruses in the field, the most practical alternative is to use transgenic plants that produce dsRNA of *ABCG4*.

## Conflict of interest

The authors declare no conflict of interest.

## Author contributions

CM conceived and supervised this research. HK and CM designed experiments. HK performed the experiments and analyzed the data. HK analyzed the NGS data. HK and CM wrote the draft manuscript and reviewed the final manuscript.

## Supporting information


**Fig. S1.** Standard curve for calculation of CMV RNA levels in CMV‐infected tobacco plants. *In vitro* transcribed CMV RNA3 was mixed with non‐inoculated tobacco total RNA (0–2 pg CMV RNA3 per 1 ng total plant RNA). qRT‐PCR was then conducted using these RNA samples. The equation for linear regression is shown. A standard curve obtaining from linear regression was used for calculating CMV RNA3 levels in Fig. 1D.
**Fig. S2.** Aphid life cycle for green and red morphs. Nymphs born from single mother aphids developed into both alate and apterous adults. The stage of nymph (N) and adult (A) stages were identified based on molting, body size and wing‐bud size as described by Jayasinghe *et al*. (2021). Scale bars: 1 mm.
**Fig. S3.** Comparison of aphid wing induction between untouched non‐inoculated and mock‐inoculated tobacco plants. The images were taken at 3 weeks after aphid placement. Total numbers of alate and apterous aphids were counted as described in Fig. 2A legend. The pie charts indicate the percentages of red/green aphids at 2 and 3 weeks after aphid placement.
**Fig. S4.** Effect of Y‐sat on *CA‐II* expression in aphids. Means (±SEM) of relative *CA‐II* expression level in alate A(2) aphids fed on CMV‐ and [CMV + Y‐sat]‐infected tobacco plants. *CA‐II*/*EF1a* mRNA levels were quantified by qRT‐PCR as described in Fig. 2C legend. Means levels for the two treatments were compared using a two‐sided Student's *t*‐test (*P < 0.05).
**Fig. S5.** Mean number of aphids per non‐inoculated, A1‐infected and A1‐ABCG4‐infected *Arabidopsis* plants after 17 days. Mean aphid numbers (*n* = 4) (±SEM) were compared for significant differences between treatments using Tukey's multiple comparison test (*P* < 0.05). Different letters above the bars indicate a significant difference between treatments.
**Fig. S6.** Percentages of sRNA reads mapped to genomes of tobacco, *Arabidopsis*, aphid and CMV. Percentages for each are given in the key to the right of each graph.
**Fig. S7.** CMV sRNA accumulation in A1‐ABCG4‐infected and uninfected Col‐0 and *dcl2/4 Arabidopsis* plants. Viral sRNAs were detected by northern blot analysis using an antisense transcript of CMV (340‐nt 3′ region of RNA3) as a probe. An ethidium bromide‐stained gel image of tRNA was included as a loading control. Note that the 24‐nt siRNA is the major sRNA in *dcl2/4* plants.
**Table S1.** Primers used in this study.

## Data Availability

All data for this study are presented in this article and Supporting Information. Further inquiries can be directed to the corresponding author (masuta@agr.hokudai.ac.jp).
