# Supplementary material for: VIGS as a strategy to reverse aphid wing induction by Y‐satellite RNA of cucumber mosaic virus
Source: FEBS Open Bio. 2023 Aug 28;13(11):2005–19. doi: 10.1002/2211-5463.13697 (PMC10626274; doi:10.1002/2211-5463.13697)
Supplement: Supplementary file 1 — Fig. S1. Standard curve for calculation of CMV RNA levels in CMV‐infected tobacco plants. In vitro transcribed CMV RNA3 was mixed with non‐inoculated tobacco total RNA (0–2 pg CMV RNA3 per 1 ng total plant RNA). qRT‐PCR was then conducted using these RNA samples. The equation for linear regression is shown. A standard curve obtaining from linear regression was used for calculating CMV RNA3 levels in Fig. 1D. Fig. S2. Aphid life cycle for green and red morphs. Nymphs born from single mother aphids developed into both alate and apterous adults. The stage of nymph (N) and adult (A) stages were identified based on molting, body size and wing‐bud size as described by Jayasinghe et al. (2021). Scale bars: 1 mm. Fig. S3. Comparison of aphid wing induction between untouched non‐inoculated and mock‐inoculated tobacco plants. The images were taken at 3 weeks after aphid placement. Total numbers of alate and apterous aphids were counted as described in Fig. 2A legend. The pie charts indicate the percentages of red/green aphids at 2 and 3 weeks after aphid placement. Fig. S4. Effect of Y‐sat on CA‐II expression in aphids. Means (±SEM) of relative CA‐II expression level in alate A(2) aphids fed on CMV‐ and [CMV + Y‐sat]‐infected tobacco plants. CA‐II/EF1a mRNA levels were quantified by qRT‐PCR as described in Fig. 2C legend. Means levels for the two treatments were compared using a two‐sided Student's t‐test (*P < 0.05). Fig. S5. Mean number of aphids per non‐inoculated, A1‐infected and A1‐ABCG4‐infected Arabidopsis plants after 17 days. Mean aphid numbers (n = 4) (±SEM) were compared for significant differences between treatments using Tukey's multiple comparison test (P < 0.05). Different letters above the bars indicate a significant difference between treatments. Fig. S6. Percentages of sRNA reads mapped to genomes of tobacco, Arabidopsis, aphid and CMV. Percentages for each are given in the key to the right of each graph. Fig. S7. CMV sRNA accumulation in A1‐ABCG4‐infected a [file FEB4-13-2005-s001.pdf]

Fig. S1

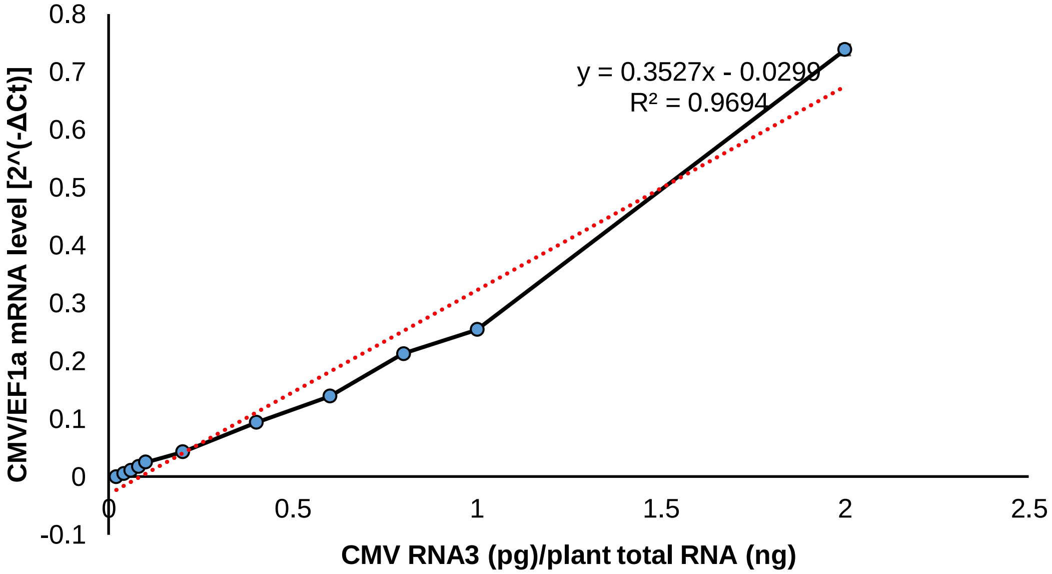
y = 0.3527x - 0.0299
R² = 0.9694
CMV/EF1a mRNA level [2^(-ΔCt)]
CMV RNA3 (pg)/plant total RNA (ng)
0.8
0.7
0.6
0.5
0.4
0.3
0.2
0.1
0
-0.1
0
0.5
1
1.5
2
2.5

Fig. S2

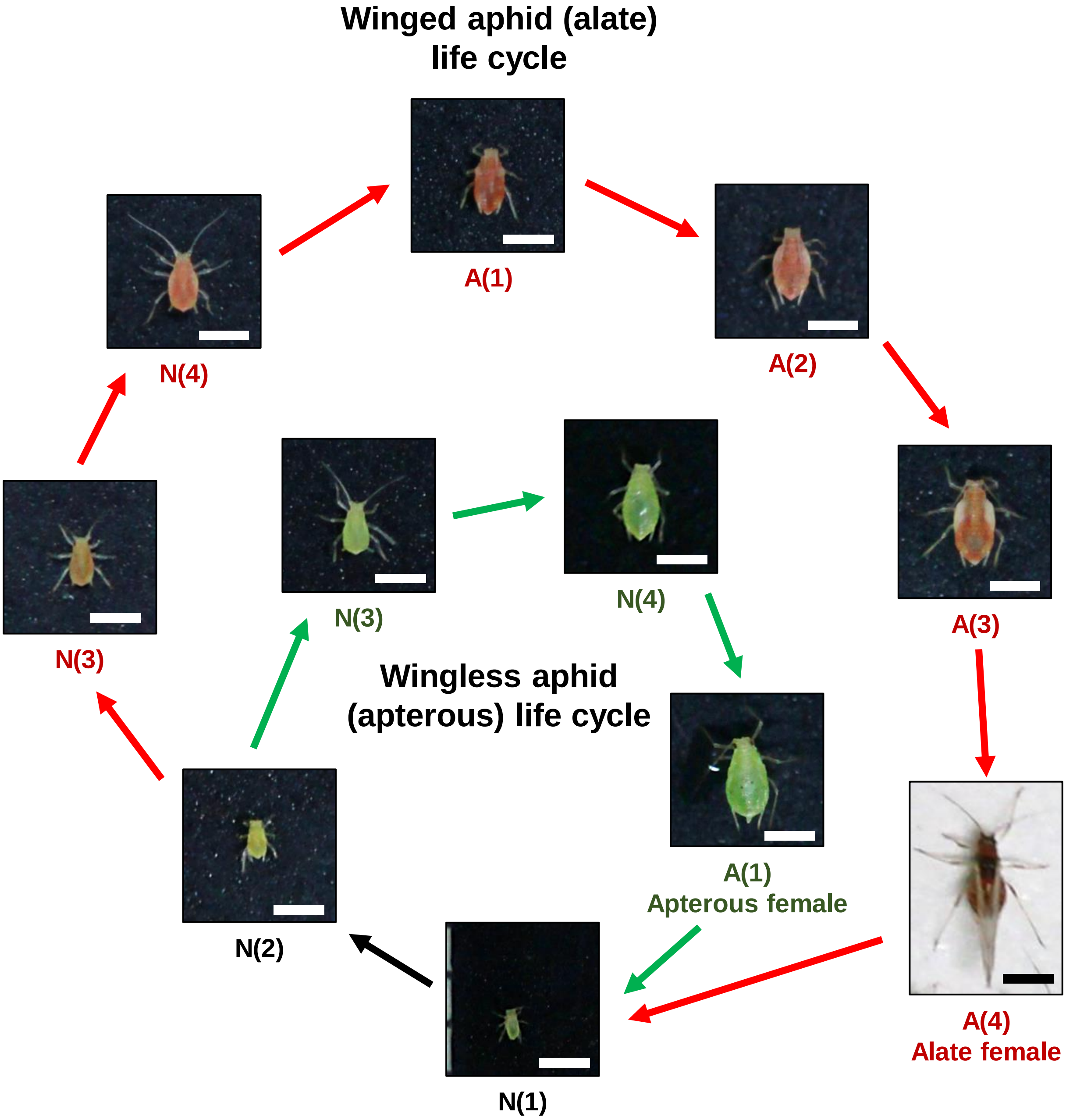
Winged aphid (alate)
life cycle
A(1)
A(2)
A(3)
A(4)
Alate female
N(4)
N(3)
N(3)
N(4)
Wingless aphid
(apterous) life cycle
A(1)
Apterous female
N(2)
N(1)

Fig. S3

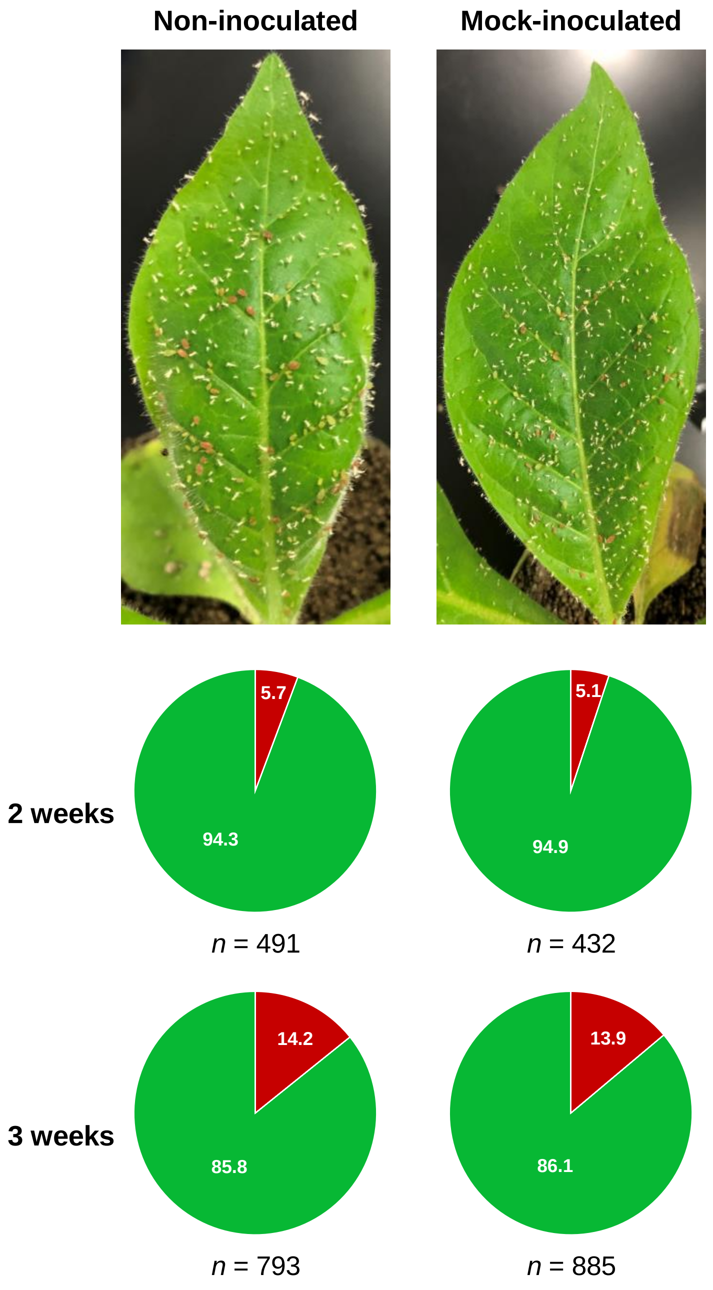

Non-inoculated
Mock-inoculated
2 weeks
5.7
94.3
n = 491
5.1
94.9
n = 432
3 weeks
14.2
85.8
n = 793
13.9
86.1
n = 885

Fig. S4

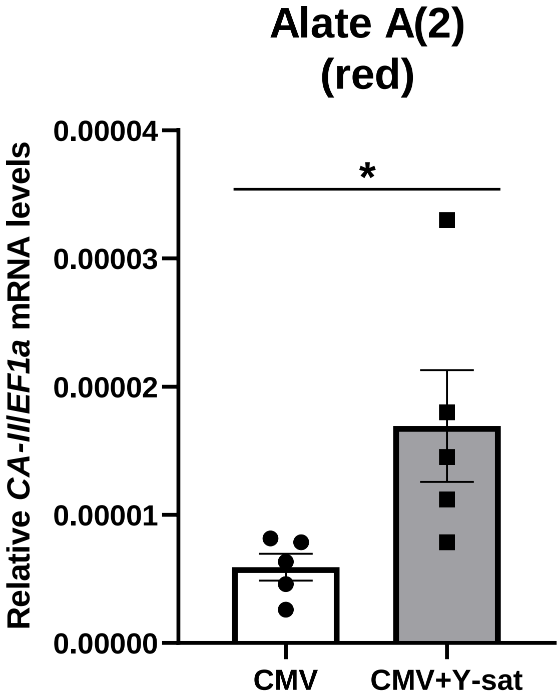
Alate A(2)
(red)
Relative CA-III/EF1a mRNA levels
0.00004
0.00003
0.00002
0.00001
0.00000
*
CMV
CMV+Y-sat

Fig. S5

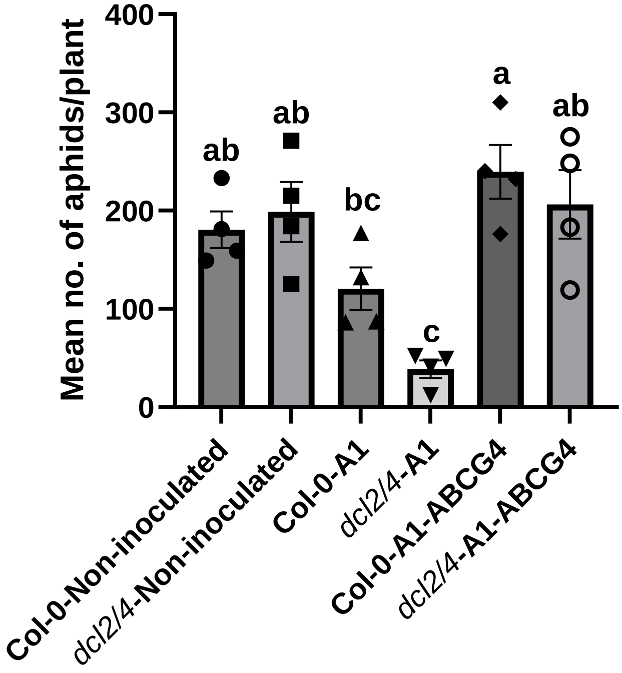

Mean no. of aphids/plant
400
300
200
100
0
ab
ab
bc
c
a
ab
Col-0-Non-inoculated
dcl2/4-Non-inoculated
Col-0-A1
dcl2/4-A1
Col-0-A1-ABCG4
dcl2/4-A1-ABCG4

Fig. S6

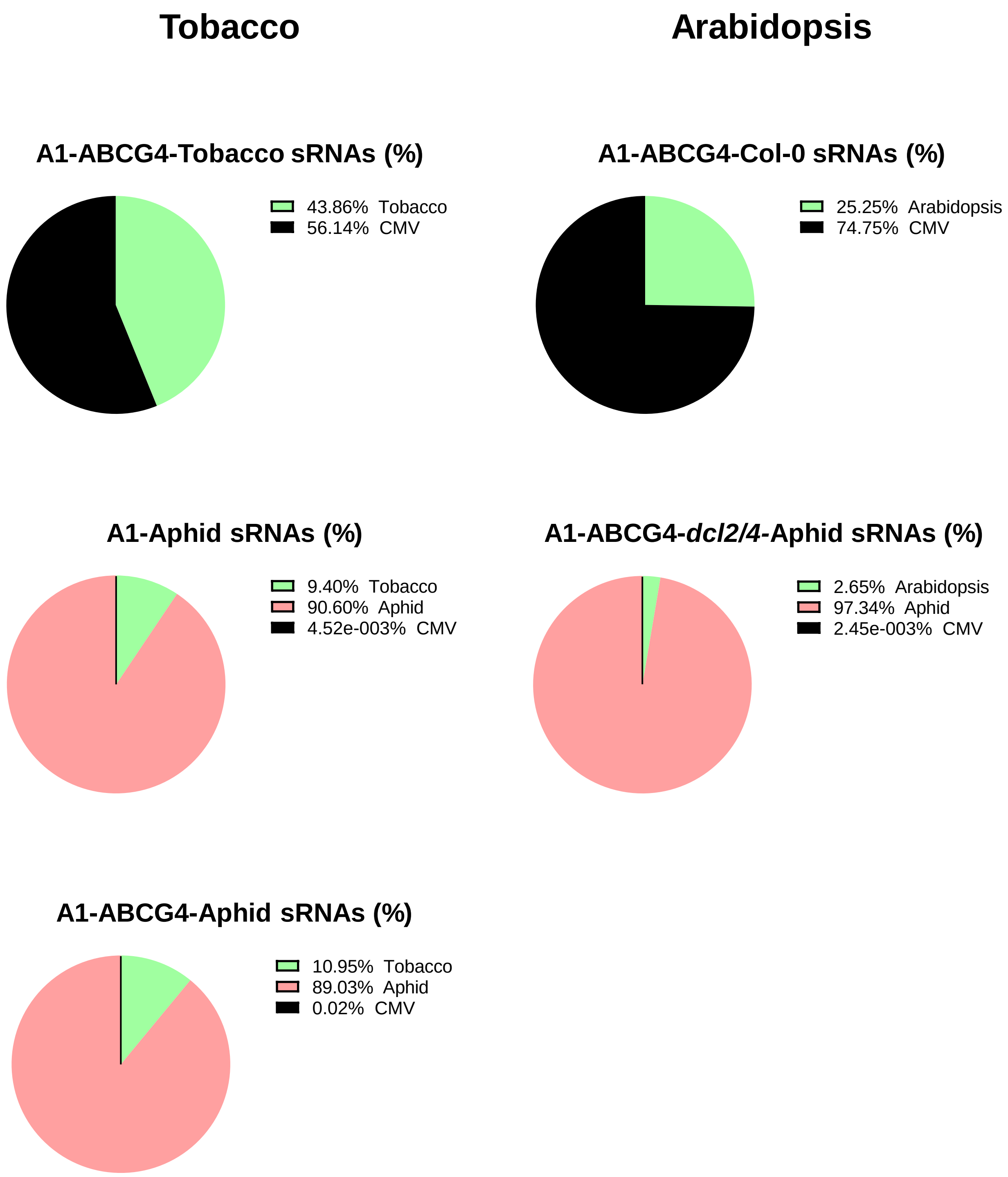
Tobacco
Arabidopsis
A1-ABCG4-Tobacco sRNAs (%)
43.86% Tobacco
56.14% CMV
A1-ABCG4-Col-0 sRNAs (%)
25.25% Arabidopsis
74.75% CMV
A1-Aphid sRNAs (%)
9.40% Tobacco
90.60% Aphid
4.52e-003% CMV
A1-ABCG4-*dcl2/4*-Aphid sRNAs (%)
2.65% Arabidopsis
97.34% Aphid
2.45e-003% CMV
A1-ABCG4-Aphid sRNAs (%)
10.95% Tobacco
89.03% Aphid
0.02% CMV

Fig. S7

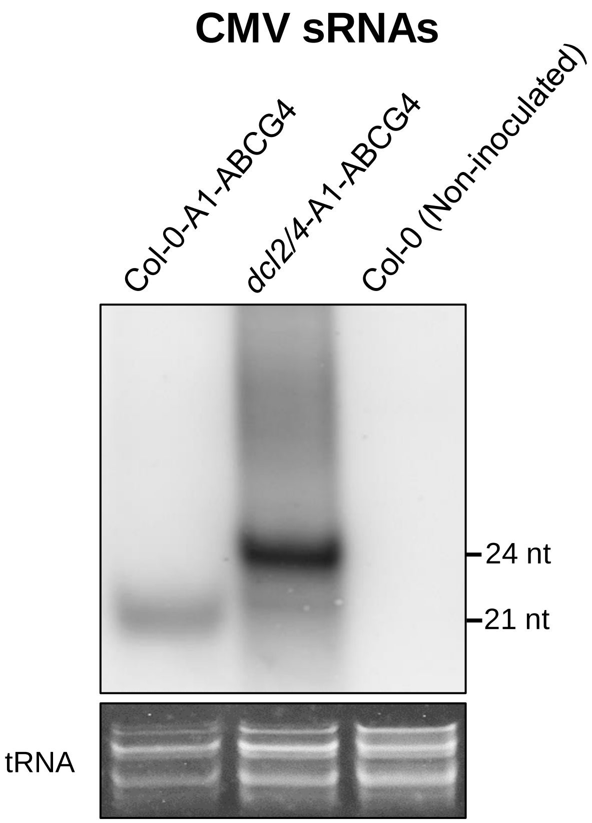
CMV sRNAs
Col-0-A1-ABCG4
dcl2/4-A1-ABCG4
Col-0 (Non-inoculated)
24 nt
21 nt
tRNA

Table S1. Primers used in this study.

Primer name	Sequence	Purpose
2b-5-up	GTACAGAGTTCAGGGTTGAGCG	Insertion check in A1 vector
R2-2814-R2	AGCAATACTGCCAACTCAGCTCC	
MpABCG4-5-190-MluI	CGCACGCGTTTTAAAGGGTGTCCGAGGGAAC	Cloning of A1-ABCG4-190
MpABCG4-3-190-StuI	GCAGGCCTCACAGCATCTTTATATTTTTGAG	
MpABCG4-5-239-MluI	CGCACGCGTAGGCTTAGACAGTTCATCATG	Cloning of A1-ABCG4-239
MpABCG4-3-239-StuI	GCAGGCCTCAAAATCAGCTGGGTTGTGATAG	
CMV-DET-5-340	GTTGACGTCGAGCACCAACGC	qRT-PCR for CMV
CMV-DET-3-340	TGGTCTCCTTTTGGAGGCCC	
T7-Y3-3	GCGTAATACGACTCACTATAGGGTGGTCTCCTT TTGGAGGCCC	Generation of probe for detection of CMV sRNAs
Q-Ap-ABCG4-5-160	AACTCCCCTGTCCCCTCTAT	qRT-PCR for *ABCG4*
Q-Ap-ABCG4-3-160	GGTGCGTCATTGATGGCTAG	
Mp-CAII-5-150	CATGTTCGTAGTTTAACGAC	qRT-PCR for *CA-II*
Mp-CAII-3-150	CCTGGGAATTGATTTGGTG	
NtEF1a-F	CCACACCTCCCACATTGCTGTCA	qRT-PCR for reference in tobacco
NtEF1a-R	CGCATGTCCCTCACAGCAAAA	
EF-1α-RT-F	GTACTTCCCAGGCCGATTGT	qRT-PCR for reference in aphid
EF-1α-RT-R	AGGTGAAGGCCAATAGAGCG	
